# A novel template-free synthesis of Na-A Zeolite for enhanced removal of heavy metals from aqueous solutions

**DOI:** 10.1371/journal.pone.0341007

**Published:** 2026-01-23

**Authors:** Mutairah S. Alshammari, Maryam AL_nosairy, Mha Albqmi, Hussein M. Ahmed, Adel M.A. Elhdad

**Affiliations:** 1 Department of Chemistry, College of Science, Jouf University, Sakaka, Aljouf, Kingdom of Soudi Arabia; 2 Housing and Building Research Center (HBRC), Sanitary and Environmental Institute. Dokki, Giza, Egypt; 3 Madinah Higher Institute of Engineering and Technology, Giza, Egypt; Universiti Teknologi Petronas: Universiti Teknologi PETRONAS, MALAYSIA

## Abstract

The removal of toxic heavy metals from wastewater remains a major environmental challenge due to their non-bio-degradability, persistence, and adverse health effects. In this work, a novel DES-assisted microwave hydrothermal route was developed for the rapid synthesis of hierarchical Na A. zeolite (NaAZ), offering a new pathway toward enhanced adsorption performance. Structural characterization (X-ray diffraction (XRD), SEM, Fourier Transform Infrared Spectroscopy” (FT-IR(Brunauer-Emmett-Teller method (BET) Scanning Electron Microscopy (SEM), Dispersive X-ray spectroscopy (EDX) confirmed the formation of highly crystalline cubic NaAZ with improved surface area and accessible porosity. Batch adsorption experiments were conducted to evaluate the uptake of Pb^2^ ⁺ , Cu^2^ ⁺ , Cd^2^ ⁺ , Ni^2^ ⁺ , and Zn^2^ ⁺ ions under varying operational conditions, including contact time, adsorbent dose, initial concentration, pH, and temperature. Results showed fast adsorption kinetics, with equilibrium reached within 90 min for most ions. Kinetic modeling revealed that the pseudo-second-order model best described the process, while equilibrium data were well-fitted to the Langmuir isotherm, indicating monolayer adsorption. Thermodynamic parameters confirmed the spontaneous and endothermic nature of the adsorption. Fixed-bed column studies demonstrated efficient dynamic adsorption, with breakthrough behavior successfully modeled using Thomas, Yoon–Nelson, and Bohart–Adams equations, confirming the material’s suitability for continuous treatment applications. Furthermore, the synthesized NaAZ exhibited excellent regeneration and reusability, maintaining high removal efficiency over multiple cycles. Compared to conventional adsorbents such as activated carbon, graphene oxide, chitosan composites, bio-char, and natural zeolite. The superior adsorption performance and distinct selectivity pattern observed for NaAZ (Pb^2^⁺ > Cu^2^⁺ > Cd^2^⁺ > Zn^2^⁺ > Ni^2^⁺) can be attributed to the synergistic effects of its hierarchical porosity and defect-engineered active sites, rather than simple ionic size or hydration radius considerations. The results indicated that the high efficiency of NaAZ was significantly for the removal of heavy metals from synthetic solutions, Overall, the optimal conditions with a contact time of 120 min, a pH of 6.0, temperature 35 ^0^C and an adsorbent dose of 250 mg.

## 1. Introduction

Environmental contamination by heavy metals such as Pb^2^ ⁺ , Cu^2^ ⁺ , Cd^2^ ⁺ , Ni^2^ ⁺ , and Zn^2^ ⁺ poses serious threats to ecosystems and human health due to their toxicity, persistence, and ability to bio-accumulate. Conventional treatment methods—chemical precipitation, ion exchange, membrane separation—are often limited by high cost, low selectivity, or secondary pollution. Cyanobacteria-based biosorbents have proven effective in heavy-metal removal Saba S. B. et al (2024) [1].

Adsorptive removal has emerged as a promising alternative, owing to its operational simplicity, regenerative potential, and effectiveness at low concentrations. Zeolites, crystalline aluminosilicates with three-dimensional microporous networks, exhibit high ion-exchange capacity, tunable surface chemistry, and excellent mechanical and chemical stability. These properties make them attractive adsorbents in diverse areas, including environmental remediation, catalysis, and separation technologies [2].

The NaAZ is among the most widely studied due to its low silica content (Si/Al ≈ 1), high cation-exchange capacity, and well-defined pore structure. Similar covalent bonding and immobilization strategies were recently developed for MOF–membrane hybrid systems Li, Z., Yu, B. et al (2025) [3]. Its efficacy in removing Pb^2^⁺ and Cu^2^ ⁺ has been demonstrated via innovative modifications. For example, a magnetic NaAZ composite with Fe₃O₄ achieved over 95% removal of Pb^2^⁺ and Cu^2^⁺ via ion exchange, while introducing magnetic separability [4]. Similarly, forming core–shell Fe₃O₄@NaA maintained high Cu^2^ ⁺ uptake (~86 mg/g), following Langmuir kinetics and demonstrating spontaneous, endothermic adsorption [5]. Biochar–clay composites have shown strong performance in cationic pollutant removal Yang, X. et al (2025) [6]. Electrocatalytic activation platforms have shown promise in improving contaminant degradation efficiency Li, Y., Bu. et al (2025) [7].

Alternative syntheses of NaAZ exploit abundant industrial by-products, advancing sustainability. NaAZ derived from fly ash significantly stabilized Pb^2^⁺ and Cd^2^ ⁺ , reducing their soil leachability and achieving stabilization rates up to 80% (Pb) and 60% (Cd) [8]. In another study, NaAZ membranes were fabricated on clay supports and achieved >99% rejection of Pb^2^⁺ and Zn^2^⁺ under pressurized filtration. Despite these advancements, key limitations persist. Conventional hydrothermal synthesis is time- and energy-intensive, while purely microporous NaAZ suffers from low diffusion rates during heavy-metal uptake especially in multicomponent matrices. Additionally, the impact of synthesis conditions on performance metrics like adsorption kinetics, selectivity, and regeneration remains underexplored [9–11]. Recent advancements in electrochemical synthesis have demonstrated dual-value-added pathways relevant to metal removal applications Wang, G. et al (2025) [12,13].

Zeolites are usually prepared from dense gels containing silica and alumina species at elevated temperatures in a relatively expensive process. This structure is responsible for its high surface area and porosity, making it an effective adsorbent. NaAZ is effective at adsorbing organic molecules and inorganic ions. They are widely used in water treatment for the removal of pollutants such as ammonium (NH₄⁺), and organic pollutants. Adsorption capacity is influenced by factors like the particle size, ionic composition, and pH of the solution. Na-zeolite can be regenerated and reused, making it a sustainable and cost-effective option for long-term use. NaAZ adsorbs various pollutants, including organic compounds, dyes, and suspended solids, due to its high surface area and micro-porous structure [14, 15]. Microwave-assisted synthesis has enabled rapid and efficient fabrication of metal–oxide composites Lu, J. et al (2024) [16].

In this work, we propose a novel, green, high-efficiency synthetic route to produce hierarchical micro–mesoporous Na A. zeolite via deep-eutectic solvent (DES)-assisted seeding and microwave hydrothermal crystallization. Our method shortens synthesis time (< 60 min), enables energy and waste reductions, and incorporates recycled aluminosilicate feed stocks to enhance sustainability. The resulting NaAZ features abundant exchangeable Na⁺ sites, tuned defect structure, and meso-porosity to improve both ion-access and heavy-metal adsorption kinetics.

We systematically evaluate its performance against Pb^2^ ⁺ , Cu^2^ ⁺ , Cd^2^ ⁺ , Ni^2^ ⁺ , and Zn^2^⁺ across equilibrium, kinetic, pH, temperature, and fixed-bed column tests, demonstrating superior capacity, selectivity, and re-generality compared to conventional NaAZ and literature benchmarks. Unlike conventional hydrothermal synthesis of NaAZ, which requires long crystallization times and high energy input, this study introduces a rapid, DES-assisted microwave hydrothermal pathway that enables the formation of highly crystalline NaAZ within significantly shorter times and with lower energy consumption. The proposed method generates a micro–mesoporous NaAZ, overcoming mass-transfer limitations of traditional microporous NaAZ. The meso-porosity enhances ion accessibility, accelerating adsorption kinetics and improving uptake efficiency for multicomponent heavy-metal systems. Previous studies mainly investigated NaAZ for single-ion systems (often Pb^2^⁺ or Cu^2^⁺). This work systematically evaluates removal of Pb^2^ ⁺ , Cu^2^ ⁺ , Cd^2^ ⁺ , Ni^2^ ⁺ , and Zn^2^**⁺** both individually and in mixture, providing insights into competitive adsorption and real wastewater applicability. Beyond batch equilibrium and kinetic studies, fixed-bed column experiments were conducted, and breakthrough data were modeled using Thomas, Yoon–Nelson, and Bohart–Adams approaches to assess dynamic adsorption capacity and mass transfer characteristics rarely reported for NaAZ. The synthesis employs green chemistry principles, including the use of deep eutectic solvents and recyclable aluminosilicate precursors, aligning the material’s development with eco-friendly and circular economy strategies. The material demonstrates high stability and retention of adsorption capacity over multiple adsorption–desorption cycles, addressing one of the key limitations of conventional adsorbents.

## 2. Materials and method

### 2.1. Synthesis of NaAZ via DES-assisted microwave hydrothermal method

A 3.0 M sodium hydroxide (NaOH, MERCK, 99.8%) solution was prepared using deionized water to provide the alkaline medium required for zeolite crystallization. Sodium meta-silicate (Na₂SiO₃, LOBA, 94%) was gradually added to the NaOH solution under continuous stirring until fully dissolved. Subsequently, aluminum sulfate (Al₂(SO₄)₃, LOBA, 99%) was introduced as the aluminum source, and the mixture was stirred thoroughly to ensure homogeneity. The resulting mixture developed into a gel-like consistency and was maintained under stirring for approximately 2 hrs to ensure complete dissolution and uniform distribution of the reactants. Na-A zeolite (NaAZ) was synthesized using a rapid, deep eutectic solvent (DES)-assisted microwave hydrothermal method. A DES was prepared by mixing choline chloride and urea in a 1:2 molar ratio under gentle heating until a clear, homogeneous liquid formed. Separately, sodium hydroxide (3.0 M) was dissolved in deionized water, followed by gradual addition of sodium metasilicate and aluminum sulfate under stirring to form a homogeneous precursor solution. The DES was then incorporated, and the resulting gel was stirred for 30 min to ensure uniformity. The mixture was transferred to a Teflon-lined microwave reactor and subjected to irradiation at 180 W and 80 °C for 45 min, enabling rapid nucleation and crystallization of NaAZ. The solid product was recovered by centrifugation, washed repeatedly with deionized water until neutral pH, and dried at 105 °C for 2–4 hrs [17]. All synthesis procedures were conducted at the Sanitary and Environmental Institute, Housing and Building National Research Center, Egypt.

### 2.2. Characterization of NaAZ

A combination of analytical techniques was employed to assess the structural, morphological, and chemical properties of the synthesized NaAZ. Surface area and pore characteristics were determined using the Brunauer–Emmett–Teller (BET) method with a Quantachrome analyzer (Quantachrome Instruments, USA). Infrared functional groups were examined using Fourier Transform Infrared Spectroscopy (FTIR) (Shimadzu FT-IR 8400S, Tokyo, Japan) within the wavenumber range of 400–4000 cm ⁻ ¹. Phase identification and crystallinity were analyzed by X-ray diffraction (XRD) using an XDS-2000 diffractometer equipped with Ni-filtered Cu Kα radiation. Surface morphology and particle shape were investigated using Scanning Electron Microscopy (SEM), while the elemental composition of the samples was evaluated through Energy Dispersive X-ray spectroscopy (EDX) using a QUANTAX system (Bruker, USA) attached to the SEM. Together, these techniques provided a comprehensive evaluation of the material’s framework structure, pore features, elemental composition, and adsorption-relevant morphology [17].

### 2.3. Chemicals and materials

Analytical-grade lead nitrate (Pb(NO₃)₂), copper sulfate pentahydrate (CuSO₄·5H₂O), cadmium nitrate tetrahydrate (Cd(NO₃)₂·4H₂O), nickel nitrate hexahydrate (Ni(NO₃)₂·6H₂O), and zinc nitrate hexahydrate (Zn(NO₃)₂·6H₂O) were purchased from Sigma–Aldrich (≥99% purity). Stock solutions (1000 mg/L) of each heavy metal were prepared using deionized water and subsequently diluted to the required concentrations [18].

### 2.4. Analytical determinations

The residual concentrations of Pb^2^ ⁺ , Cu^2^ ⁺ , Cd^2^ ⁺ , Ni^2^ ⁺ , and Zn^2^⁺ in the aqueous solution were determined using Atomic Absorption Spectroscopy (AAS, PerkinElmer Analyst 400) calibrated with external standards. Blanks and spiked recovery samples were included to ensure data accuracy. Perform blank experiments to check for metal loss due to container adsorption. Run adsorption tests in triplicate. Use procedural blanks and standard solutions to validate Atomic absorption performance [19].

### 2.5. Batch adsorption experiments

Batch adsorption studies were-performed to evaluate the removal of Pb^2^ ⁺ , Cu^2^ ⁺ , Cd^2^ ⁺ , Ni^2^ ⁺ , and Zn^2^ ⁺ ions from aqueous solutions. Precisely weighed masses of NaAZ (0.010, 0.015, 0.020, 0.025, 0.030, 0.035, 0.040, 0.045, and 0.050 g) were-added separately to 100 mL of metal ion solution in 250 mL Erlenmeyer flasks. Initial concentrations of the metal ions (C₀) ranged from 0.5, 1.0, 1.5, 2.0, 2.5, 3.0, 3.5, 4.0, 4.5, and 5.0 mg**/**L.

The aqueous solution of samples were-incubated in a thermostatic orbital shaker at 25 ± 2 °C and 150 rpm for contact times of 10, 20, 30, 40, 50, 60, 90, 120, 150, and 180 min to determine adsorption kinetics and equilibrium behavior. At each time interval, samples were-withdrawn filtration through 0.45 µm cellulose nitrate membranes.

The effect of pH on the removal of Pb^2^ ⁺ , Cu^2^ ⁺ , Cd^2^ ⁺ , Ni^2^ ⁺ , and Zn^2^⁺ in the solution has been established and considered an important parameter affecting the performance of the adsorption process. At varied pH levels (3, 6, and 9), removing of Pb^2^ ⁺ , Cu^2^ ⁺ , Cd^2^ ⁺ , Ni^2^ ⁺ , and Zn^2^⁺ from the aqueous solution was conducted with a constant dosage of 0.025 g at 150 rpm/90 min, and 0.5 mg/L. Equilibrium thermodynamics (ΔG°, ΔH°, ΔS°), Temperatures: 15, 25, 35, 45 °C. Conditions: fixed adsorbent dose (0.025 g), pH (6.0), time (90) and C₀ (0.5 mg/L). t = t_eq_ at each T. Equations (1, 2) were using to compute distribution constant. Dimension less by using concentration units consistently or by dividing by standard state) [20].


$${\text{K}}_{\text{c}}={\text{q}}_{\text{e}}/{\text{C}}_{\text{e}}{\text{K}}_{\text{c}}$$ (1)

$${\text{InK}}_{\text{c}}=-{\Delta \text{H}}^{\circ }/\text{R}\;\cdot \text{1}/\text{T}\;+\;{\Delta \text{S}}^{\circ }/\text{R}$$ (2)

Plot the ln K_c_ vs 1/T to extract ΔH° (slope) and ΔS° (intercept). 1/T to extract: ΔG° negative → spontaneous; ΔH° positive → endothermic; negative → exothermic.

### 2.6. Adsorption isotherm study

Equilibrium adsorption isotherms were-investigated by varying the initial concentrations of Pb^2^ ⁺ , Cu^2^ ⁺ , Cd^2^ ⁺ , Ni^2^ ⁺ , and Zn^2^ ⁺ ions. For each experiment, 0.025 g of NaAZ was added to 100 mL of metal ion solution with initial concentrations (C_0_) ranging from 0.5 mg**/**L. The suspensions were-incubated at 25 ± 2 °C on an orbital shaker 150 rpm for a contact time of 90 min, which was previously determined as sufficient to reach adsorption equilibrium. Equations (3, 4) were used to determine the amount of pollutants adsorbed at equilibrium (q_e_) and the percentage removed (R %). Fit data to Langmuir, Freundlich, and Temkin, and models. Model fitting and parameter estimation were-performed by the coefficient of determination (R^2^) [21,22].


$$\text{R}\%=\frac{\left(Ci-Ce\right)}{Ci}\;x\;\text{1}\text{0}\text{0}\;$$ (3)

$$\text{qe}=\frac{(Ci-Ce)V}{W}\;$$ (4)

Where q_e_ (mg/g) represents the amount of pollutants adsorbed at equilibrium, V (L) is the volume of the solution, W (g) is the mass of the nanoparticles used, C_i_, and C_e_ (mg/L) represents Pb^2^ ⁺ , Cu^2^ ⁺ , Cd^2^ ⁺ , Ni^2^ ⁺ , and Zn^2^ ⁺ ions concentrations at initial and equilibrium conditions, respectively.

#### 2.6.1. Langmuir isotherm model.

Adsorption isotherms, which are typically the ratio between the amount adsorbed and that was left in solution at equilibrium at a specific temperature, are used to describe equilibrium studies that give the capacity of the adsorbent and adsorbate [21]. The Langmuir model is predicated on the hypothesis that maximal adsorption happens in the presence of a saturated monolayer of solute molecules on the adsorbent surface, the adsorption energy is constant, and there is no adsorbate molecule migration in the surface plane. The Langmuir isotherm model implies that physical factors drive monolayer sorption. The Langmuir isotherm is given by Equations (5–7):


$$\text{qe}=\frac{\text{C}.\text{K}\;\text{qmax}}{\text{1}+\text{KL}}\;\;\;\;\;\;\;\;\;\;\;\;\;\;\;\;\;\;\;\;\;\;\;\;\;\;\;\;\;\;\;\;\;\;\;\;\;\;\;$$ (5)

$$\frac{\text{Ce}}{\text{qe}}=\frac{\text{1}}{\text{qmax}\;\;.\;\text{KL}}+\frac{\text{Ce}}{\text{qmax}}\;\;\;\;\;\;\;\;\;\;\;\;\;\;\;\;\;\;\;\;\;\;\;$$ (6)

$$\text{RL}=\frac{\text{1}}{\text{1}+\text{Co}.\text{KL}}\;\;\;\;\;\;\;\;\;\;\;\;\;\;\;\;\;\;\;\;\;\;\;\;\;\;\;\;\;\;\;\;\;\;\;\;\;\;\;$$ (7)

where q_max_ and K are the Langmuir constants, where qe is the dose of Pb^2^ ⁺ , Cu^2^ ⁺ , Cd^2^ ⁺ , Ni^2^ ⁺ , and Zn^2^ ⁺ ions adsorbed on a specific dose of adsorbent (mg/g), C_e_ is the equilibrium concentration of the solution (mg/L) and q_max_ is the maximum dose of Pb^2^ ⁺ , Cu^2^ ⁺ , Cd^2^ ⁺ , Ni^2^ ⁺ , and Zn^2^ ⁺ ions concentration required to form a monolayer (mg/g). The values of q_m_ and K can be determined from the linear plot of C_e_/q_e_ versus C_e_ [22]. R_L_ is separation factor calculated through Langmuir model.

#### 2.6.2. Freundlich isotherm model.

The Freundlich isotherm model proposes that many sites with various adsorption energies are involved in the empirical relationship that describes the adsorption of solutes from a liquid to a solid surface. The system’s characteristics K_F_ and n are the indicators of the adsorption capacity and adsorption intensity, respectively. The Freundlich model’s capacity to match the experimental data was-investigated. The intercept value of K_F_ and the slope of n were-calculated for this scenario using the plot of log C_e_ vs. log q_e_. The Freundlich isotherms appear when the surface is heterogeneous and the absorption is multilayered and bound to sites on the surface [22].. The Freundlich isotherm is-given by Equation (8):


$$\text{log}\text{qe}=\text{log}\text{KF}+\frac{\text{1}}{\text{n}}\text{log}\text{Ce}\;\;\;\;$$ (8)

where K is the Freundlich equilibrium constant (mg/g), 1/n = Intensity parameter C_e_ = Equilibrium concentration of adsorbate, q_e_ is the dose of solute adsorbed.

#### 2.6.3. Temkin isotherm.

Equation (9) were used to determine the (adsorbate–adsorbent interactions and linear heat of adsorption):


qe= B lnKt.Ce  (9)

Where B = RT/b, with R being the universal gas constant (8.314 J/mol.K), T the absolute temperature (K), b the Temkin constant related to the heat of adsorption (J/mol), and K_T_ (L/mg) the Temkin isotherm constant [21,22].

### 2.7. Adsorption Kinetic Study

Kinetic experiments were-conducted to investigate the adsorption rate and mechanism of Pb^2^ ⁺ , Cu^2^ ⁺ , Cd^2^ ⁺ , Ni^2^ ⁺ , and Zn² ⁺ ions onto **NaAZ**. For each test, known masses of NaAZ (0.025 g) were added to 100 mL of metal ion solutions with initial concentrations (C_0_ 0.5 mg/L). The suspensions were agitated in an orbital shaker at 25 ± 2 °C and 150 rpm. Samples were-withdrawn at predetermined time intervals (10, 20, 30, 40, 50, 60, 90, 120, 150, and 180 min) to monitor the adsorption process. The adsorption capacity at time t (qt, mg/g) was calculated using Equation (2). It was possible to characterize the kinetics for each adsorbent, using pseudo-first, pseudo-second-order kinetic models, and and Intraparticle diffusion model (Weber–Morris).The pseudo-first-order kinetics follows the Lagergren model expressed by Equation (10):


$$\text{Log}\left(q_{eq}-q_{t}\right)=\text{log}q_{eq}-K_{\text{1}}.\;\;\;t/\text{2}.\text{303}$$ (10)

where q_t_ is the adsorbed dose of Pb^2^ ⁺ , Cu^2^ ⁺ , Cd^2^ ⁺ , Ni^2^ ⁺ , and Zn^2^ ⁺ ions (mg/g) in t time (min) and k_1_ is the pseudo-first-order constant (min^-1^).

Through linear and angular constant of log graphic (q_eq_ – q_t_) in the function of time, q_eq_ and k_1_ can be calculated, respectively. Comparing the experimentally obtained values for q_eq_ calculated by pseudo- second-order kinetics by Equation (11):


$$t/q_{t}=\;1/K_{\text{2}}q_{{eq}^{\text{2}\;}}+t/q_{eq}$$ (11)

where k_2_ is the pseudo-second-order constant (g/mg. min) obtained by calculation of linear coefficient and q_eq_ is calculated through angular coefficient [23].

To evaluating the goodness of fit for non-linear kinetic models (pseudo-first-order, and pseudo-second) in adsorption and biodegradation processes, error analysis is essential such as sum of Squared Errors (SSE). Equation (12) [24].


$$\begin{array}{c} \text{SSE}=\;\sum {\mathrm{\backslash nolimits}}_{i=\text{1}}^{n}.\;({\text{q}}_{\text{e}}\text{exp}-{\text{q}}_{\text{e}}\;\text{cal}{)}^{\text{2}} \end{array}$$ (12)

Where q_e, exp_, is the experimental adsorption capacity and q_e_, _calc_ is the calculated value from the model. Lower SSE values indicate a better fit of the model to the experimental data, and highly sensitive to large deviations.

The experimentally obtained values for q_eq_ calculated by Intraparticle diffusion model by Equation (13):


$$q_{t}=K_{\text{\{ID}}\}\;\text{\{T}\}\;{\;}^{0.5}+\text{c}$$ (13)

where k_id_ (mg/g·min ⁻ ^1^/^2^) is the intraparticle diffusion rate constant and C (mg/g) represents the boundary layer effect.

### 2.8 Fixed-bed column tests

Fixed-bed column experiments were-performed to evaluate the dynamic performance of NaAZ for simultaneous removal of Pb^2^ ⁺ , Cu^2^ ⁺ , Cd^2^ ⁺ , Ni^2^⁺ and Zn^2^ ⁺ .

#### 2.8.1. Column packing and operating conditions.

A glass column (internal diameter = 10 mm, i.d. = 1.0 cm) was wet-packed with NaAZ to a bed height (**L**) of 10.0 cm. Prior to testing, the packed bed was conditioned with deionized water until the effluent conductivity matched the influent. The feed solution consisted of an equimolar mixture of the five metal ions at C_0_ = 5.0 mg**/**L each. The feed was introduced to the column in down flow mode at a constant volumetric flow rate Q = 2.6 mL**/**min using a peristaltic pump. All tests were-performed at ambient laboratory temperature (≈25 °C). Effluent samples were collected at regular time intervals (Hussein et al 2022) [25]. (Table 1) show Column geometric parameters. Effluent was-sampled at fixed intervals (2–5 min). The concentration ratio C_t_/C_0_ was plotted versus time to generate breakthrough curves. Breakthrough time (b_t_) was defined at C_t_/C_0_ = 0.10 (10% of influent) and full breakthrough at C_t_/C_0_ = 0.90.

**Table 1:** Column geometric parameters.

Radius r = i.d/2 = 0.50 cm	0.50 cm
Cross-sectional-area A = πr^2^ = π(0.50)^2^	0.785 cm^2^
Bed-volume Vb = A × L = 0.785 cm^2^ × 10.0 cm = 7.85 cm^3^	7.85 mL
Empty-bed-contact-time (EBCT) =V_b_/Q = 7.85 mL/2.6 mL\min^− 1^	3.02 min

#### 2.8.2. Bohart-Adams model.

The nonlinear form of Bohart-Adams models is shown in Equation (14). In fixed-bed column, it is employed to determine the initial condition of the surface operation. The model is based on the surface reaction theory. The adsorption rate is considered to be proportional to both the residual adsorbent capacity and the initial concentration of the adsorbed.


$$\frac{Cads}{Co}\;=\frac{\text{1}}{\text{1}+e^{\left(\propto -B+t\right)}}\;$$ (14)

Where, 𝛼 = 𝑘_𝐵𝐴_*𝑁_𝑜_*𝑍, 𝛽 = 𝑘_𝐵𝐴_ * 𝐶_𝑜_ * 𝑡. K_BA_ signifies the Bohart-Adams kinetic constant (L/mg. min). 𝑁_𝑜_ and Z is the saturation concentration (mg/L) as well as the bed depth of the column (cm), respectively [26,27].

#### 2.8.3. Yoon-Nelson model.

It is used to describe the time of column run before the replacement of column or regeneration became mandatory. Yoon-Nelson model assumes that, the rate of decrease in the probability of adsorption for each adsorbate molecule is proportional to the probability of adsorbate adsorption and the probability of adsorbate breakthrough on the adsorbent. The nonlinear form of the model is given in Equation (15). The Yoon-Nelson model is a descriptive model that uses experimental data to calculate parameters that are then entered into the model [26,27].


$$\text{Log}\;\text{t}\;=\;{\text{k}}^{\prime }\;–\;\text{a}\;\text{Log}\;\text{C1}\;$$ (15)

t = breakthrough time (min), t = 50% contaminant breakthrough time (min)

k’ = rate constant (min^-1^)

C_I_ = contaminant assault concentration mg/L

#### 2.8.4. Thomas model.

Thomas model is widely used to describe the column performance and to predict the breakthrough curves. The nonlinear form of the model is represented in Equation (16) [26,27].


$$\frac{Cads}{Co}\;=(\frac{Kth*qth*m}{Q}-\text{Kth}*\text{Co}*\text{t})$$ (16)

𝑘_𝑇ℎ_ represents the Thomas constant in (mL/min.mg), 𝑞_𝑇ℎ_ denotes the uptake capacity calculated by the Thomas model in (mg/g), m denotes the adsorbent mass in the column in (g), and Q represents the volume flow rate in (mL/min).

### 2.9. Regeneration and reusability studies of NaAZ for removal of heavy metal

Regeneration of NaAZ is essential to restore its adsorption capacity after saturation and to enable repeated use in wastewater treatment. Reusability not only improves cost-effectiveness but also enhances the sustainability of the treatment process. Previous investigations have shown that regenerated NaAZ can efficiently remove heavy metal ions such as Pb^2^ ⁺ , Cu^2^ ⁺ , Cd^2^ ⁺ , Ni^2^ ⁺ , and Zn^2^⁺ across multiple adsorption cycles. During operation, NaAZ gradually becomes saturated with adsorbed ions, leading to diminished removal efficiency. Regeneration reverses this process by desorbing the retained ions and restoring active exchange sites. Among the available techniques, both chemical and thermal regeneration are commonly applied. Thermal regeneration (calcination) involves heating the exhausted material to around 600 °C to decompose and remove adsorbed organic matter. Although this method can achieve near-complete regeneration and is effective for organics and volatile contaminants, it is energy-intensive and less practical for large-scale use dominated by inorganic pollutants. Chemical regeneration is more suitable for heavy metal-loaded zeolite. In this approach, the exhausted NaAZ is treated with an acid solution—typically hydrochloric acid (HCl, 1.0 N) which replaces the retained metal ions with sodium or hydrogen ions. Following regeneration, the material is thoroughly washed with deionized water to remove excess acid and prevent secondary contamination.

In this study, NaAZ was subjected to five consecutive adsorptions–desorption cycles to assess its regeneration potential. After each cycle of heavy metal adsorption, the material or column bed was flushed with 1.0 N HCl to restore ion-exchange capacity. The regenerated zeolite was then reused under the same experimental conditions. The multi-cycle evaluation allowed the determination of adsorption capacity retention, efficiency loss, and structural stability over repeated use. These findings are critical for evaluating the economic feasibility of NaAZ in real wastewater treatment applications and determining its long-term performance under operational conditions. we have described the equipment used for the adsorption–desorption cycles, including the regeneration vessel, temperature control system, agitation conditions, and the procedure for monitoring each regeneration cycle

## 3. Results and discussion

### 3.1. Characterization of NaAZ

#### 3.1.1. Physical properties and BET analysis of NaAZ.

**Fig 1:**
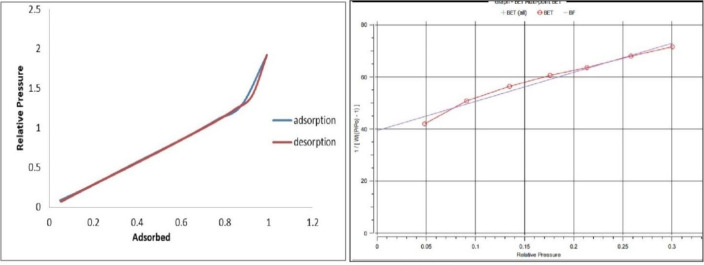
BET analysis of NaAZ.

The NaAZ due to its aluminosilicate framework and sodium-rich composition, exhibits high cation-exchange capacity and notable ionic mobility. These characteristics make it suitable for applications such as ion exchange, adsorption, gas separation, water treatment, desiccation, and detergent formulation. To examine its textural features, nitrogen adsorption–desorption measurements were performed using the BET method. The results confirmed that the material contains a uniform microporous network with a considerable specific surface area and well-defined pore architecture. These structural traits enable efficient interaction with dissolved species, offering multiple accessible active sites for adsorption. The presence of interconnected pores and a large surface-to-volume ratio enhances the material’s ability to capture contaminants including metal ions, ammonium, and various organic pollutants, as illustrated in Fig 1 and summarized in Table 2. The BET-derived values for surface area and pore volume provide useful indicators of the adsorbent’s functionality, accessibility of internal channels, and overall suitability for water purification and related environmental applications [28].

**Table 2:** Physical properties and BET analysis of NaAZ.

Appearance	Orange
Smell	None
Plasticity	None
Humidity at drying 110 °C	10.3
Solubility in water	None
pH value	6.5–7.5
Density	2.23 g.cm^-3^
Molecular Weight	28.0134 g
Specific surface area (BET)	22.98 m^2^/g
Average Pore radius	2.8952 nm
Cross Section Area	16.2 Å²/molec

#### 3.1.2. X-Ray diffraction (XRD).

**Fig 2:**
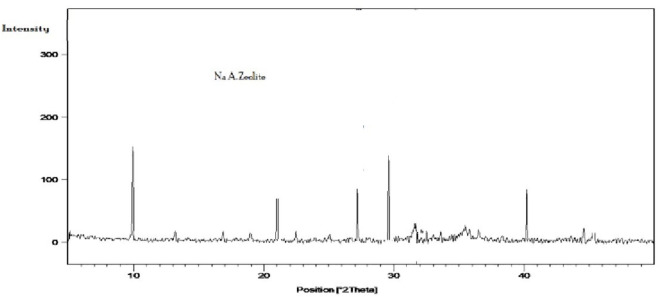
X. ray diffraction patterns of NaAZ.

The X-ray diffraction (XRD) profile of the synthesized NaAZ, illustrated in Fig 2, displays prominent reflections at 2θ values of 10.22°, 21.74°, 27.08°, 30.16°, and 40.88°. These peaks correspond to the (100), (200), (321), (400), and (420) crystallographic planes, which are characteristic of the LTA zeolite structure. The intensity and sharpness of the peaks, combined with the minimal amorphous background signal, demonstrate that the material is well crystallized and of high phase purity [29]. The diffraction pattern confirms that NaAZ is the dominant crystalline phase. However, very weak signals attributable to hydroxysodalite were detected when higher NaOH concentrations (3.0 N) were used during synthesis. This observation agrees with previous investigations, which noted that elevated alkalinity can promote hydroxysodalite formation at the expense of pure LTA phases.

Elemental composition determined by X-ray fluorescence (XRF) is summarized in Table 3. The primary constituents were silicon (≈65 wt%), aluminum (≈17.5 wt%), and sodium (≈6.5 wt%), matching the stoichiometry typically reported for NaAZ based on its aluminosilicate network. The calculated SiO₂/Al₂O₃ ratio was approximately 3.7, which is consistent with the range reported in earlier studies, including the work of Zhang et al. (2019) [30]. We now explicitly state the NaOH concentration used in the optimized synthesis of the novel NaAZ. The final synthesis was carried out using 2.0 N NaOH, a condition under which no detectable hydroxysodalite peaks were observed in the XRD profile. As noted, hydroxysodalite formation occurred only when higher alkalinity (3.0 N NaOH) was tested during preliminary trials; however, this condition was not employed in the final synthesis reported in this study. We have clarified in the text that the optimized 2.0 N NaOH condition yielded phase-pure, highly crystalline LTA-type NaAZ with no measurable hydroxysodalite impurities. Accordingly, the adsorption results reported in the manuscript represent the performance of pure NaAZ, free from secondary phases. In addition, we added a brief discussion of the potential implications of hydroxysodalite formation. Although the impurity appeared only under non-optimized conditions, we now note that hydroxysodalite typically possesses lower surface area and reduced pore accessibility compared to LTA zeolite.

**Table 3:** Chemical composition of the NaAZ.

Chemical composition	Amount %
SiO_2_	65
Al_2_O_3_	17.5
Na_2_O	6.5
Others	11

#### 3.1.3. Scanning electron microscopy (SEM).

**Fig 3:**
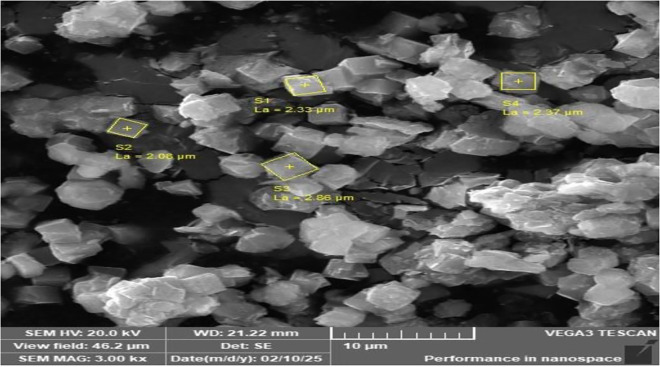
SEM micrographs of NaAZ.

Fig 3 presents the SEM micrographs of the synthesized NaAZ, which clearly display well-defined, uniformly distributed cubic crystals with sharp edges. The average particle size is observed to be below 70 µm. The images also show aggregates of partially reacted meta-kaolinite plates interspersed with discrete NaAZ cubic particles, confirming the successful conversion of the precursor material into the zeolitic phase. The SEM observations provide critical insight into the surface morphology, particle size distribution, and textural features of the synthesized material—factors that significantly influence adsorption performance. Smaller crystal dimensions are generally associated with increased external surface area, which facilitates enhanced adsorption capacity and faster uptake of solutes. High-resolution SEM further reveals cubical particles ranging from 1 µm to 3 µm in size, consistent with the typical morphology of NaAZ reported by Cheng et al. (2018) [31]. The observed uniformity in particle size supports stable and predictable adsorption behavior and improves handling characteristics in potential industrial applications. Complementary BET surface analysis confirmed the coexistence of both micropores and mesopores, indicating that the adopted synthesis approach successfully produced hierarchical porosity without compromising the intrinsic microporous framework of NaAZ. This observation aligns with findings by Wang et al. (2020) [32]. The presence of mesopores is particularly advantageous, as it enhances intra-particle diffusion, increases accessibility to active sites, and contributes to the improved adsorption kinetics recorded for the synthesized zeolite.

#### 3.1.4. Energy dispersive X-ray spectroscopy (EDX).

**Fig 4:**
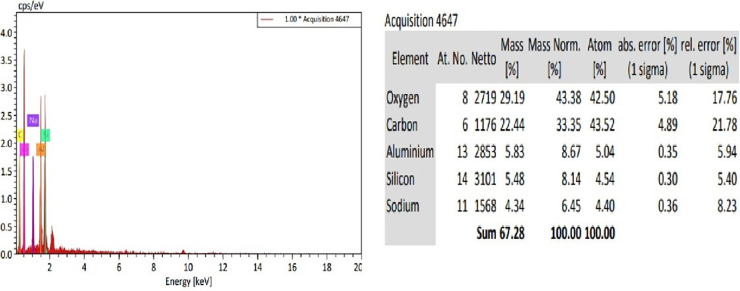
EDX and percentage constituents of NaAZ.

The EDX spectroscopy was performed to verify the elemental composition of the synthesized NaAZ and to confirm the successful formation of the aluminosilicate framework. The EDX spectrum Fig 4 clearly demonstrates the presence of sodium (Na), aluminum (Al), silicon (Si), and oxygen (O), which are the key constituents of NaAZ. The measured elemental distribution aligns closely with the theoretical stoichiometry expected for NaAZ, validating the structural integrity and successful synthesis of the material. Quantitatively, the NaAZ sample was found to contain approximately 29.19 wt% oxygen, 5.48 wt% silicon, 5.83 wt% aluminum, and 4.34 wt% sodium. These values correspond well with the characteristic composition of Na-A zeolite and reflect an appropriate Si/Al ratio, which is critical for determining ion-exchange capacity and adsorption behavior. Zeolitic materials typically comprise a framework of tetrahedrally coordinated aluminum and silicon linked by oxygen atoms, with charge-balancing cations such as sodium residing within the pore structure. In some cases, other cations such as titanium, tin, or zinc may substitute or coexist depending on the synthesis conditions and precursor composition [33]. The absence of extraneous elements in the spectrum further suggests high material purity and the effective transformation of the starting material into the target zeolitic phase.

#### 3.1.5. Fourier transform infrared spectroscopy (FT-IR).

**Fig 5:**
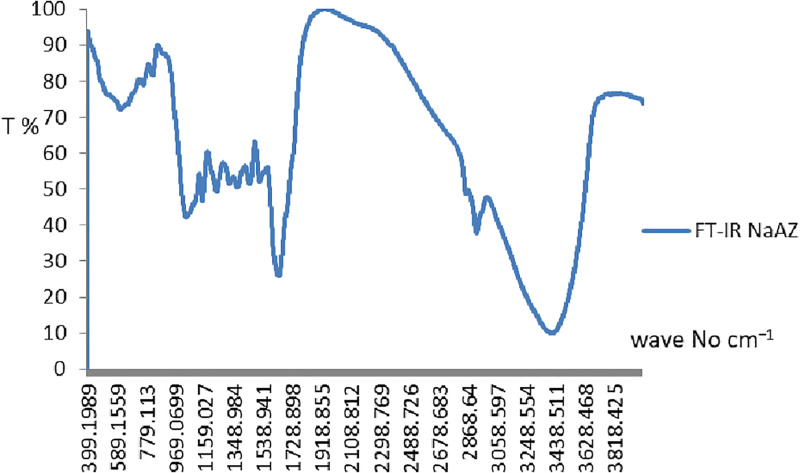
FT-IR for the NaAZ.

FTIR spectroscopy was conducted to verify the functional groups and vibrational modes associated with the aluminosilicate framework of the synthesized NaAZ. The spectrum Fig 5 displays key absorption bands at 3421, 1166, and 1080 cm ⁻ ¹, which are characteristic of Si–O and Al–O stretching vibrations within the zeolitic lattice. The broad band centered around 3421 cm ⁻ ¹ corresponds to O–H stretching from surface hydroxyl groups and physisorbed water, suggesting the retention of moisture within the pore channels. A strong absorption peak at 1080 cm ⁻ ¹ is associated with Si–O stretching and reflects contributions from the meta-kaolinite precursor. Notably, the disappearance of the band typically observed near 530 cm ⁻ ¹, attributed to Al[O(OH)]₆ bending, confirms the dehydroxylation of kaolinite during calcination and its subsequent structural reorganization into the zeolite framework [34,35]. Residual water-related bands are attributed to van der Waals interactions between hydroxyl groups and the electrostatic field generated by Na⁺ cations within the structure [36]. Additionally, the breathing mode of the double six-ring (D6R) units, reported to occur near 530 cm ⁻ ¹ in LTA-type zeolites [36,37], provides further evidence of the successful formation of the target topology. Overall, the FT-IR results verify the development of a well-defined aluminosilicate framework with high structural order and purity, consistent with the excellent adsorption characteristics reported for NaAZ materials [38].

### 3.2. Effect of contact time

**Fig 6:**
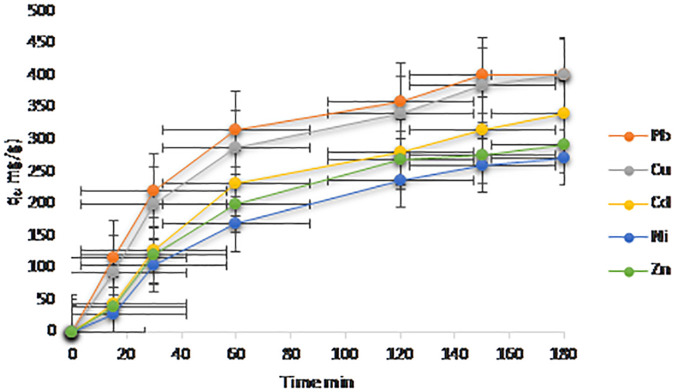
A adsorption capacity (qₜ, mg/g) vs. time (min).

The adsorption kinetics of Pb^2^ ⁺ , Cu^2^ ⁺ , Cd^2^ ⁺ , Ni^2^ ⁺ , and Zn^2^⁺ onto the hierarchical NaAZ displayed a characteristic two-stage behavior. An initial rapid uptake occurred within the first 30–60 min, followed by a slower adsorption phase that gradually approached equilibrium. The equilibrium times were approximately 120 min for Pb^2^ ⁺ & Cu^2^⁺ and about 150 min for Cd^2^ ⁺ , Ni^2^ ⁺ , and Zn^2^ ⁺ . This biphasic pattern is typical for ion-exchange adsorbents, where the early rapid stage corresponds to the occupation of abundant as shown in Fig 6, readily accessible exchangeable Na⁺ sites on the external surface of the zeolite particles [39]. The slower phase is dominated by intra-particles diffusion, involving transport of metal ions into micro-pores and meso-pores within the zeolite framework [40]. The hierarchical structure synthesized in this study, featuring enhanced meso-porosity and improved pore connectivity, significantly accelerated the adsorption process, reducing equilibrium times. This improvement is attributed to the facilitated diffusion pathways provided by meso-pores, which reduce internal mass transfer resistance and enable faster access of metal ions to internal adsorption sites [41]. Enhanced mass transfer kinetics contribute to more efficient heavy metal removal, particularly important for practical applications where shorter treatment times are desirable [42]. Dual-modification strategies have been used to enhance stability and charge behavior in zinc-ion systems Zhang, M. et al (2025) [43].

### 3.3. Effect of adsorbent dose

**Fig 7:**
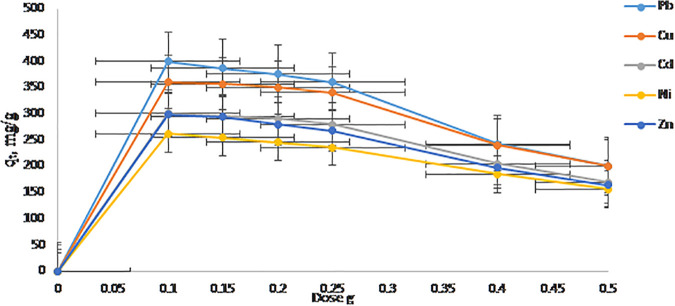
A adsorption capacity (qₜ, mg/g) vs. dose(g).

The removal efficiency of Pb^2^⁺ and Cu^2^ ⁺ ions increased consistently with increasing adsorbent dose, ranging from 100 mg to 500 mg, achieving over 95% removal at the highest dose of 500 mg as shown in Fig 7. This trend is attributed to the increased availability of active adsorption sites with higher amounts of zeolite, which enhances the likelihood of metal ion capture from solution [44]. However, despite this increase in removal efficiency, the adsorption capacity (q_e_, mg/g) decreased at higher adsorbent doses. This decrease can be explained by the presence of unsaturated binding sites when excessive adsorbent is added, leading to inefficient utilization of the adsorbent surface [38]. Additionally, partial aggregation of adsorbent particles at higher doses can reduce the effective surface area accessible to metal ions, further limiting adsorption capacity per unit mass. An optimal adsorbent dose of 250 mg was identified as a balance between maximizing removal efficiency and ensuring effective adsorbent utilization. Selecting this dose avoids excessive material use and minimizes issues related to particle aggregation while maintaining high adsorption performance [42].

### 3.4. Effect of initial concentration

**Fig 8:**
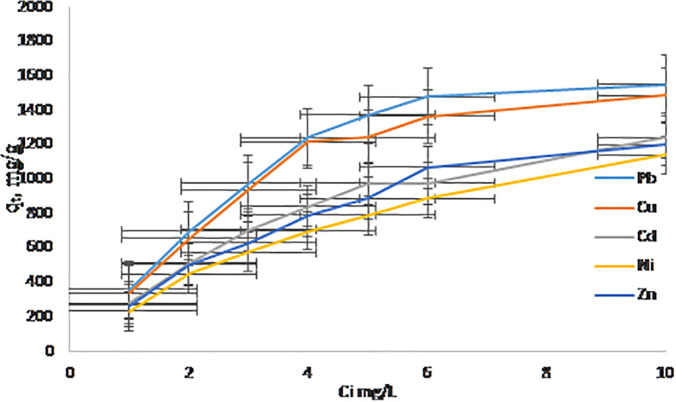
A adsorption capacity (qₜ, mg/g) vs. initial concentration mg/L.

At a fixed adsorbent dose and contact time (250 mg 120 min), the adsorption capacity (qₑ) of Pb^2^ ⁺ , Cu^2^ ⁺ , Cd^2^ ⁺ , Zn^2^ ⁺ , and Ni^2^ ⁺ increased with rising initial metal ion concentration (C_₀_) as shown in Fig 8. This behavior is typical in adsorption processes, where higher concentrations provide a greater driving force for mass transfer, leading to enhanced loading of adsorbate on available adsorption sites [44]. However, the removal efficiency (R %) exhibited a decreasing trend at higher C_₀_ values, which can be attributed to the saturation of adsorption sites. As these sites become fully occupied, the proportion of metal ions removed relative to the initial amount declines, despite the increase in absolute uptake capacity. Isotherm analysis further clarified the affinity sequence for these metal ions on the hierarchical NaAZ, Pb²⁺ > Cu²⁺ > Cd²⁺ > Zn²⁺ > Ni² ⁺ . This order is consistent with their hydrated ionic radii and established ion-exchange selectivity of NaAZ frameworks, where smaller or less hydrated ions tend to have lower affinity due to weaker electrostatic interactions [41]. Pb²⁺ and Cu² ⁺ , possessing favorable ionic sizes and hydration characteristics, exhibited the strongest affinity, which corresponds well with their higher maximum adsorption capacities reported in this study.

### 3.5. Effect of pH

**Fig 9:**
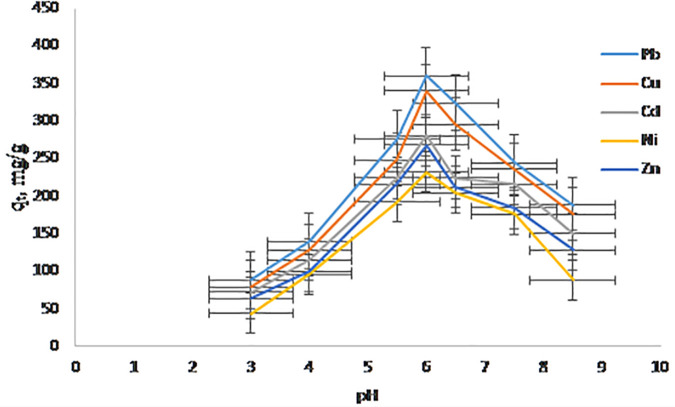
A adsorption capacity (qₜ, mg/g) vs. pH values.

The pH is the most critical parameter affecting any adsorption studies due to their interference in the solid–solution interface, affecting the charges of the active sites of the adsorbents and the Pb^2^ ⁺ , Cu^2^ ⁺ , Cd^2^ ⁺ , Ni^2^ ⁺ , and Zn^2^ ⁺ behavior in the solution. The adsorption of Pb^2^ ⁺ , Cu^2^ ⁺ , Cd^2^ ⁺ , Ni^2^ ⁺ , and Zn^2^⁺ onto hierarchical NaAZ was found to be highly pH-dependent. At low pH values (3–4) as shown in Fig 9, the adsorption capacity was significantly reduced due to the competitive adsorption between abundant protons (H⁺) and metal ions for the negatively charged sites on the zeolite surface. This proton competition effectively inhibits metal ion uptake, consistent with previous studies on ion-exchange materials [44]. The optimal adsorption occurred in the pH range of 5.5–6.5, which coincides with the predominant presence of free divalent metal cations in solution and a negatively charged zeolite surface. Under these conditions, ion exchange between Na⁺ ions and metal ions is facilitated, leading to maximum adsorption efficiency [42]. The negative surface charge enhances electrostatic attraction, promoting stronger interactions with metal cations. Above pH 7, a slight decrease in dissolved metal concentrations was observed, likely due to the onset of metal hydroxide precipitation rather than true adsorption. This was confirmed through control experiments without adsorbent, which showed similar reductions in metal concentration, indicating precipitation rather than adsorption [40]. Therefore, pH values above 7 are not optimal for adsorption studies of these metals as precipitation can interfere with adsorption measurements. Overall, the optimal pH window of 5.5–6.0 balances the availability of free metal ions and the surface charge characteristics of the zeolite, maximizing ion-exchange efficiency and removal capacity.

### 3.6. Effect of temperature

**Fig 10:**
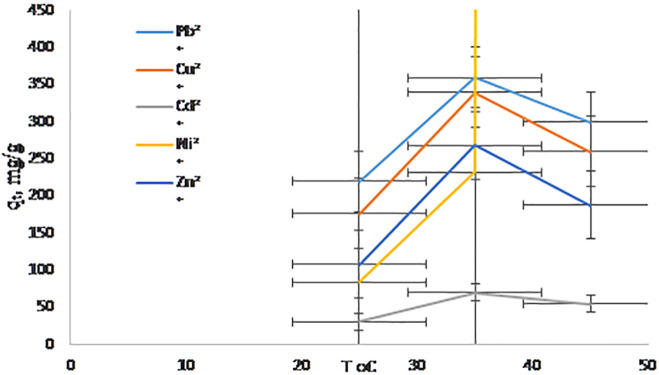
A adsorption capacity (qₜ, mg/g) vs. T ^o^C.

The adsorption capacity of Pb^2^ ⁺ , Cu^2^ ⁺ , Cd^2^ ⁺ , Zn^2^ ⁺ , and Ni^2^⁺ on hierarchical NaAZ increased with rising temperature (15–45 °C), as shown in Fig 10, indicating that the process is endothermic. Higher temperatures provide the energy needed to overcome activation barriers associated with chemisorption and enhance ion mobility within the porous structure, promoting stronger interactions between metal ions and the zeolite framework [44].

Thermodynamic analysis further supports this conclusion. The negative Gibbs free energy values (ΔG° = −10 to −20 kJ·mol ⁻ ¹) confirm that adsorption is spontaneous under the studied conditions, with larger negativity at higher temperatures reflecting increasingly favorable uptake [45]. Positive enthalpy changes (ΔH° = 20–35 kJ·mol ⁻ ¹) corroborate the endothermic nature of the process and indicate that chemisorption—including ion exchange or surface complexation—dominates over physical adsorption mechanisms [41]. Importantly, the positive entropy changes (ΔS°) reflect increased randomness at the solid–solution interface. This is primarily due to the partial release of structured water molecules from the hydration shells of metal ions as they bind to active sites on the zeolite surface. The resulting increase in disorder contributes favorably to the overall thermodynamic driving force, reinforcing the spontaneity of the adsorption process and highlighting the dynamic nature of the interface during ion exchange. Together, these thermodynamic parameters confirm that the hierarchical NaAZ facilitates spontaneous, endothermic chemisorption accompanied by increased system disorder, enhancing the efficiency of heavy-metal removal.

### 3.7. Isotherm studies

**Fig 11:**
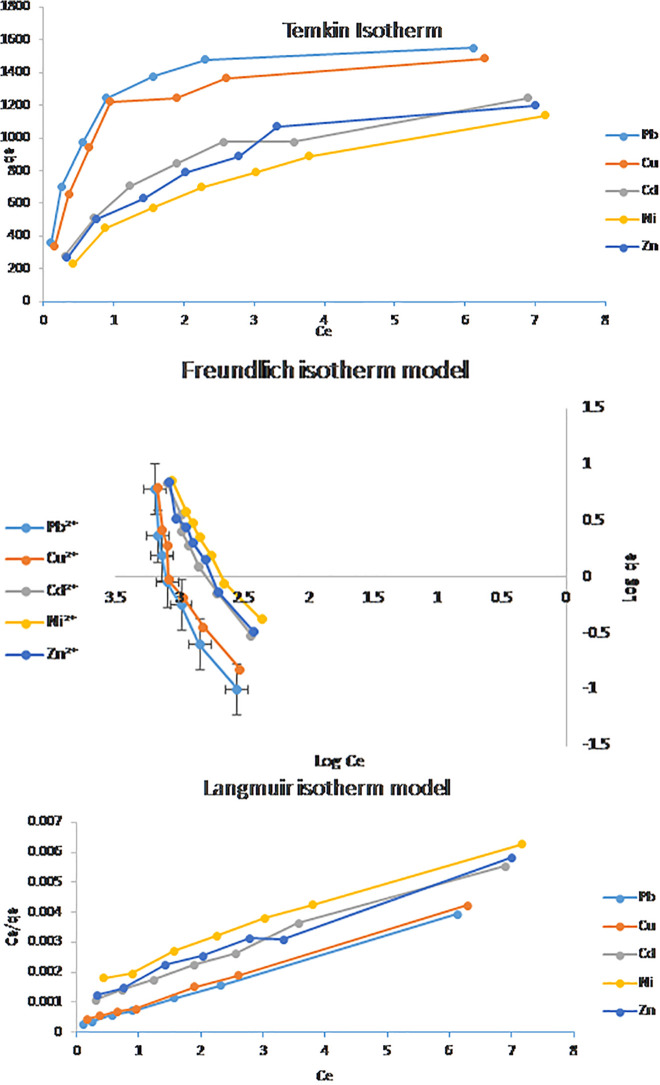
Langmuir, Temkin and Freundlich isotherm models for metal ions on NaAZ.

Fig 11 and Table 4 present the equilibrium adsorption data for Pb^2^ ⁺ , Cu^2^ ⁺ , Cd^2^ ⁺ , Zn^2^ ⁺ , and Ni^2^ ⁺ ions onto the hierarchical NaAZ. The results show that the Langmuir isotherm model provides the best fit for all metal ions, with correlation coefficients R^2^ > 0.98. This excellent agreement indicates monolayer adsorption on homogeneous active sites, where each ion occupies a discrete adsorption site without interaction with neighboring species. The Langmuir parameters revealed a clear capacity trend of Pb^2^⁺ > Cu^2^⁺ > Cd^2^⁺ > Zn^2^⁺ > Ni^2^ ⁺ , consistent with ion-exchange–dominated monolayer adsorption. The superior adsorption performance and distinct selectivity pattern of NaAZ stem from the combined effects of hierarchical porosity and defect-engineered active sites rather than simple ionic size or hydration radius considerations. The DES-assisted microwave synthesis promotes the formation of interconnected micro–mesoporous channels, which enhances ion diffusion and facilitates rapid access to the internal LTA cages. This structural hierarchy particularly benefits larger and more polarizable ions such as Pb^2^ ⁺ , enabling deeper penetration and more efficient utilization of internal adsorption sites. that equilibrium was reached after 120 min. Additionally, the controlled incorporation of framework defects—such as oxygen-rich coordination sites (Si–O⁻ and Al–O⁻), hydroxyl bridges, and charge-compensating Na⁺ centers—creates highly reactive Lewis basic sites capable of forming strong inner-sphere complexes. Pb^2^ ⁺ , being softer and more polarizable, shows the highest affinity for these defect sites, resulting in the highest q_max. Cu^2^ ⁺ follows due to its ability to form stable surface complexes and partially dehydrate at the interface. In contrast, Zn^2^⁺ and Ni^2^ ⁺ exhibit weaker interaction due to their higher charge density and stronger hydration shells, which limit dehydration and penetration into the LTA pores.

**Table 4:** The isotherm adsorption parameters for the studied metal ions adsorbed on NaAZ.

Langmuir	q_max_	K	R^2^
Pb²⁺	1866	3.0	0.9995
Cu²⁺	1666	2.0	0.9983
Cd²⁺	1428	0.77	0.9931
Ni²⁺	1228	0.43	0.9905
Zn²⁺	1144	0.67	0.9892
Freundlich	n	K	R^2^
Pb²⁺	0.416667	> 1000	0.8734
Cu²⁺	0.454545	> 1000	0.848
Cd²⁺	0.5	> 1000	0.9507
Ni²⁺	0.526316	> 1000	0.9596
Zn²⁺	0.588235	> 1000	0.9715
Temkin Isotherm	b	K	R^2^
Pb²⁺	3.026846	>1000	0.5098
Cu²⁺	3.316984	>1000	0.5525
Cd²⁺	5.368369	>1000	0.8006
Ni²⁺	7.44393	>1000	0.8965
Zn²⁺	5.969026	>1000	0.8065

Although the Freundlich constants (n < 1.0) indicate non-favorable adsorption on heterogeneous sites, the superior fit of the Langmuir model confirms that monolayer ion exchange is the dominant mechanism. Overall, these findings demonstrate that the hierarchical NaAZ synthesized in this study exhibits excellent adsorption capacities, well-defined selectivity, and strong potential for heavy-metal remediation applications [44,45].

### 3.8. Kinetic studies

**Fig 12:**
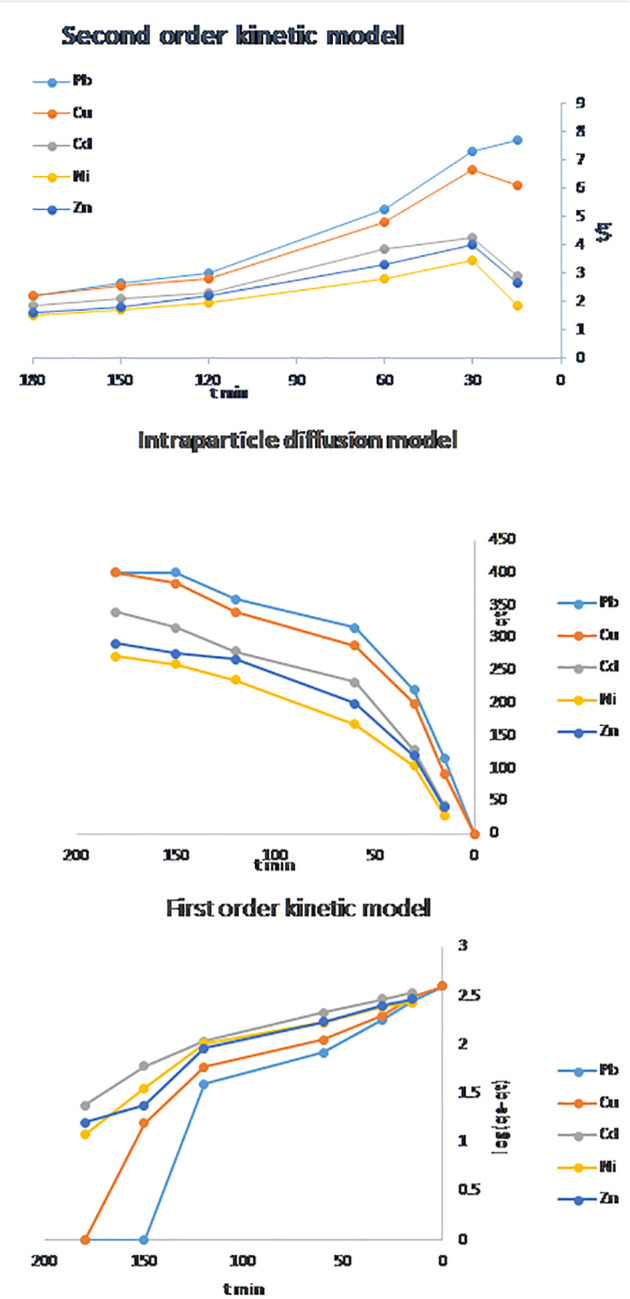
Kinetic models for metal ion adsorbed on NaAZ.

The kinetic behavior of Pb^2^ ⁺ , Cu^2^ ⁺ , Cd^2^ ⁺ , Ni^2^ ⁺ , and Zn^2^ ⁺ adsorption onto the hierarchical NaAZ was analyzed using the pseudo-first-order, pseudo-second-order, and intra-particle diffusion models. As presented in Fig. 12 and Table 5, the pseudo-first-order model produced relatively low correlation coefficients (R² = 0.8302–0.9081), indicating that it does not adequately describe the adsorption kinetics. In contrast, the pseudo-second-order model yielded markedly higher R^2^ values (0.9335–0.9676) along with consistent rate constants (K₂ = 1.84 × 10 ⁻ ⁵–1.56 × 10 ⁻ ⁴ g mg ⁻ ¹ min ⁻ ¹), confirming that this model best represents the experimental data. The strong agreement with the pseudo-second-order model indicates that chemisorption—through electron sharing or exchange between framework Na⁺ ions and divalent metal cations—is the dominant rate-controlling mechanism [44].

**Table 5:** The adsorption kinetic models parameters regarding the of metals ions adsorbed on NaAZ.

kinetic models	metal	R^2^	K
**First**	pb	0.9043	0.0149
	Cu	0.8686	0.0121
	Cd	0.8302	0.0114
	Ni	0.8992	0.0112
	Zn	0.9081	0.119
Second	pb	0.9491	0.000156
	Cu	0.9335	0.000113
	Cd	0.9662	3.54E-05
	Ni	0.9463	1.84E-05
	Zn	0.9676	3.49E-05
Intra-particle diffusion model (Weber–Morris)	Pb	0.8099	1.957
	Cu	0.857	1.9
	Cd	0.8882	1.61
	Ni	0.9076	1.37
	Zn	0.8703	1.4

The adsorption process proceeds through a characteristic two-stage pathway. The initial rapid uptake within the first 30–60 minutes is attributed to surface adsorption onto abundant, easily accessible exchange sites. This is followed by a slower stage in which metal ions diffuse into the micro- and mesoporous regions of the zeolite. Such sequential behavior is typical for porous adsorbents and highlights the critical role of pore architecture in influencing adsorption kinetics [45].

Further examination using the intra-particle diffusion (Weber–Morris) model resulted in lower R^2^ values (0.8099–0.9076) and non-zero intercepts, demonstrating that intra-particle diffusion contributes to, but does not solely control, the overall adsorption process. The diffusion rate constants (K_id_ = 1.37–1.957 mg g ⁻ ¹ min ⁻ ⁰·⁵) support the presence of a two-step mechanism: rapid surface adsorption followed by slower diffusion into the hierarchical micro–mesoporous network. The enhanced porosity generated through DES-assisted synthesis significantly improves mass transfer compared to conventional microporous NaA zeolites, enabling faster adsorption kinetics [42]. This structural advantage is especially important for practical water treatment applications where rapid removal of heavy metals is required [46–47].

In summary, the kinetic and diffusion analyses highlight the combined effects of chemisorption and improved pore accessibility in achieving the superior adsorption performance of the hierarchical NaA zeolite toward divalent heavy metals [48–50].

### 3.9. Fixed-bed column studies

**Fig 13:**
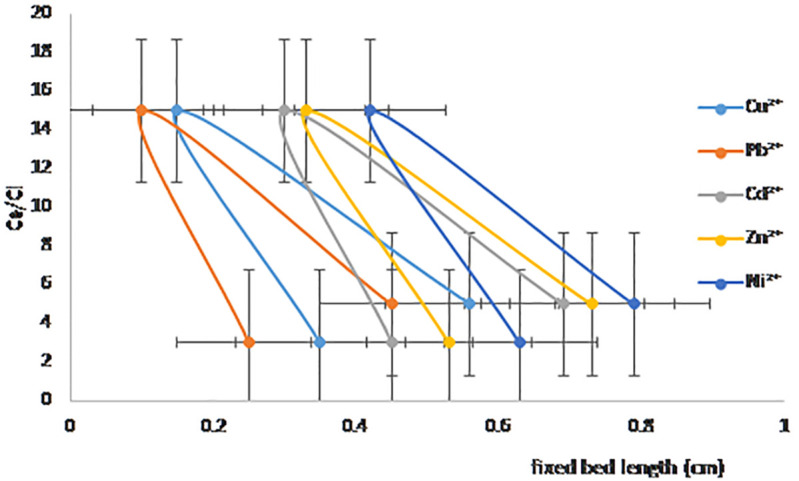
Fixed-bed column studies for metal ion adsorbed on NaAZ.

Dynamic adsorption studies based on the DD were conducted to evaluate the effect of the pre-selected three independent variables on the adsorption capacity of Pb^2^ ⁺ , Cu^2^ ⁺ , Cd^2^ ⁺ , Zn^2^ ⁺ , and Ni^2^ ⁺ adsorption onto NaAZ. The Fixed-bed length (3, 5, 15) cm, effluent Flow rate (Q) (2 mL min^−1^) and initial concentration of metal (1, 5, 10) mg/L were chosen as the independent variables. The combined effect of bed height (3, 5, 15) cm and initial concentration (1, 5, 10) mg/L on the adsorption capacity of Pb^2^ ⁺ , Cu^2^ ⁺ , Cd^2^ ⁺ , Zn^2^ ⁺ , and Ni^2^⁺ at a volumetric constant Flow rate (2 mL min^−1^) are shown in Table 6. The response function (the adsorption capacity of Pb^2^ ⁺ , Cu^2^ ⁺ , Cd^2^ ⁺ , Zn^2^ ⁺ , and Ni^2^ ⁺ increased with an increasing initial concentration of ions as well as the bed height. This trend could be explained by the fact that increasing the bed height results in an increased availability of active sites for adsorption and a higher influent concentration provides a higher driving force for transfer to overcome mass-transfer resistance. The Fig 13 show relation between C_e_/C_i_ against to fixed bed length (cm), The breakthrough curves from the fixed-bed column experiments exhibited notably steeper fronts for Pb² ⁺ , Cu² ⁺ , Cd^2^ ⁺ , Zn^2^ ⁺ , and Ni^2^ ⁺ adsorption onto NaAZ indicating their higher affinity and faster adsorption kinetics. This behavior aligns with their higher ion-exchange selectivity and smaller hydrated ionic radii, which facilitate quicker diffusion and stronger interaction with the NaAZ adsorption sites [42]. The estimated mass transfer zone (MTZ) length of approximately 3, 5, 15 cm indicates efficient utilization of the adsorbent bed, suggesting that the hierarchical pore structure of the NaAZ enhances intra-particle diffusion and reduces resistance to mass transfer. Such structural advantages contribute to shorter equilibrium times and improved dynamic capacities compared to conventional zeolites [45]. Collectively, these findings demonstrate the superior performance of hierarchical NaAZ for continuous-flow heavy metal removal. The combination of high affinity for Pb²⁺ and Cu² ⁺ , favorable kinetic behavior, and efficient bed utilization highlights its practical potential for industrial wastewater treatment applications [51–53].

**Table 6:** Experimental data in the DD for removal of metals ions.

fixed bed length (cm)	3, 5, 15
Flow rate (Q, mL min^− 1^)	2.0
Initial metals concentration (C_0_, mg/L)	1, 5, 10

### 3.10. Mathematical modeling of breakthrough curves

**Fig 14:**
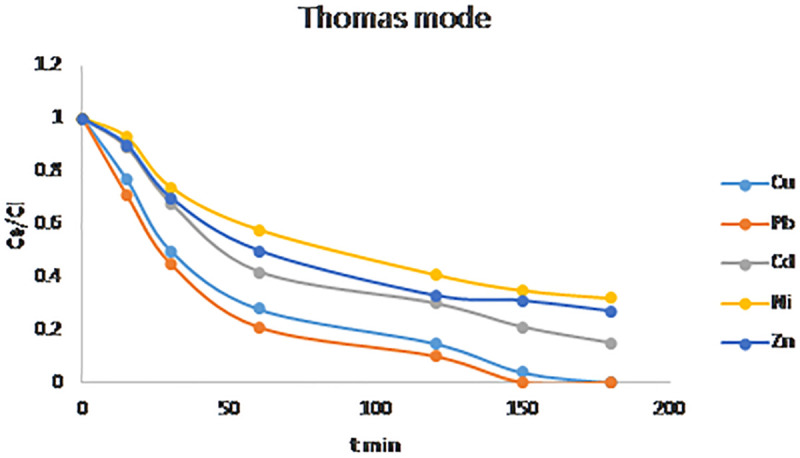
Thomas models breakthrough curves for phenol adsorption.

**Fig 15:**
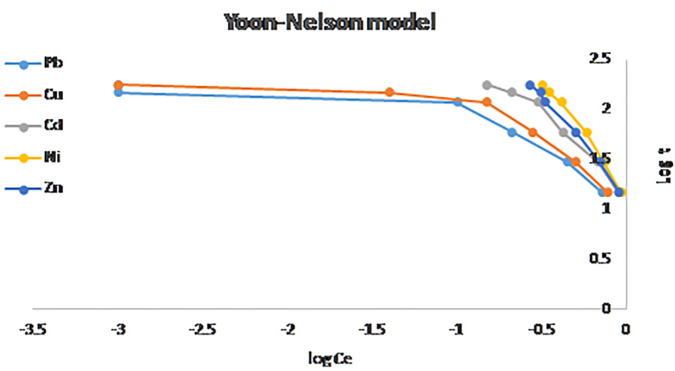
Yoon–Nelson models breakthrough curves for phenol adsorption.

**Fig 16:**
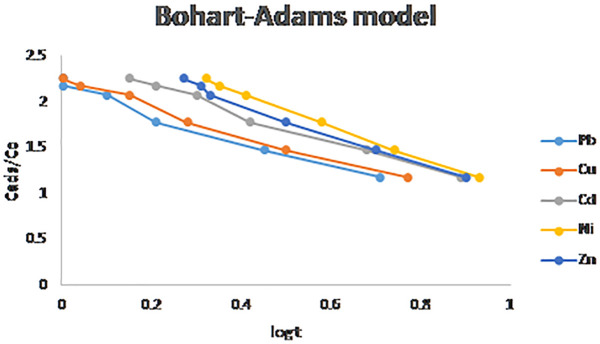
Bohart-Adams models breakthrough curves for phenol adsorption.

Removal of inorganic and organic matters from wastewater was performed by fixed bed adsorption. To study the performance of fixed bed column for phenol removal by adsorption process, eight analytical models were validated. Breakthrough curves were calculated for Bed Depth Service Time, Thomas, Yoon–Nelson, and Bohart–Adams equations as shown in Fig 14–16. the data obtained revealed that the experimental data fits and respond well to each correlation coefficient values as shown in Table 7, the Bohart–Adams model demonstrated the best correlation with the experimental data, exhibiting an R^2^ value is 0.9787, 0.9889, 0.9917, 0.9989, and 0.9927, for Pb, Cu, Cd, Ni, and Zn respectively [54–56]. This excellent agreement indicates that the Bohart–Adams model accurately describes the adsorption kinetics and breakthrough characteristics of fixed-bed column [57,58].

**Table 7:** The mathematical modeling parameters regarding the of metals ions adsorbed on NaAZ.

Metal ions	Thomas model	Yoon–Nelson models	Bohart-Adams model
Pb	0.8099	0.9178	0.9787
Cu	0.857	0.7209	0.9889
/Cd	0.8966	0.6515	0.9917
Ni	0.9186	0.7863	0.9989
Zn	0.8846	0.8993	0.9927

### 3.11. Regeneration and reusability

**Fig 17:**
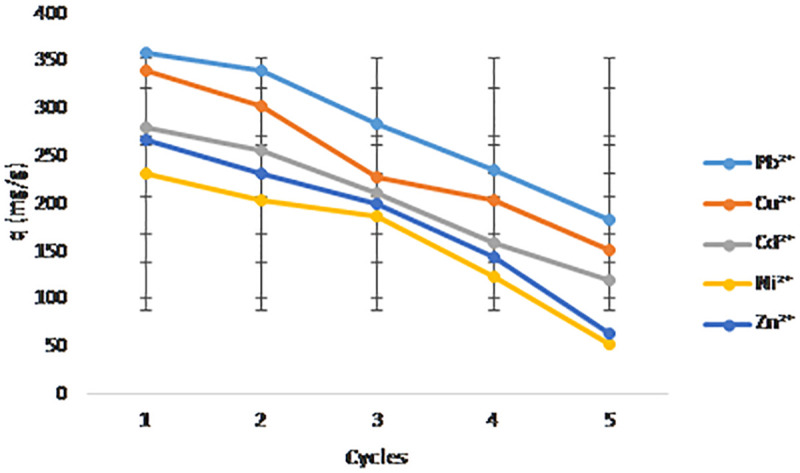
Regeneration study for metal ion adsorbed on NaAZ.

The hierarchical NaAZ exhibited excellent regeneration performance, (Fig 17) showing the five consecutive adsorption–desorption cycles as the re-generation process (360, 340, 280, 232, and 268) mg/g for Pb^2^ ⁺ , Cu^2^ ⁺ , Cd^2^ ⁺ , Ni^2^ ⁺ , and Zn^2^ ⁺ ions, respectively. after These results demonstrate the potential of the synthesized zeolite for cost-effective and sustainable application in continuous heavy metal removal processes. The regeneration and reusability results demonstrate that the hierarchical NaAZ possesses excellent stability and sustained adsorption performance for the removal of Pb^2^ ⁺ , Cu^2^ ⁺ , Cd^2^ ⁺ , Ni^2^ ⁺ , and Zn^2^ ⁺ ions over multiple adsorption–desorption cycles. After five consecutive cycles, the zeolite retained high efficiency for removal for all metals, with 90%, 85%, 70%, 58%, and 67% for Pb^2^ ⁺ , Cu^2^ ⁺ , Cd^2^ ⁺ , Ni^2^ ⁺ , and Zn^2^ ⁺ ions, respectively. This indicates strong binding affinity and efficient desorption using 0.1 M HCl as the re-generator. The slight decrease in adsorption capacity across cycles can be attributed primarily to incomplete desorption of the metal ions and possible minor fouling or pore blockage, consistent with previous studies on zeolite regeneration [44]. Notably, the retention trend across metals correlates well with their initial adsorption capacities and affinities Pb^2^⁺ and Cu^2^ ⁺ maintain higher capacities due to their stronger ion-exchange interactions and smaller hydrated ionic radii [42]. Cd^2^ ⁺ , Ni^2^ ⁺ , and Zn^2^ ⁺ exhibit slightly lower retention, potentially because of their comparatively weaker interactions with the zeolite surface. The practical implication of th/ese findings is significant, the NaAZ synthesized via the hierarchical method offers a robust adsorbent suitable for repeated use in real wastewater treatment systems, reducing operational costs associated with adsorbent replacement. Furthermore, the effective regeneration with a relatively mild regenerate (HCl) demonstrates an environmentally friendly approach to adsorbent reuse [45]. Overall, the balance between high initial adsorption capacity, strong affinity for heavy metals, and excellent regeneration performance confirms the potential of this hierarchical NaAZ as a promising material for sustainable and cost-effective heavy metal remediation.

### 3.12. Comparison of NaAZ with other adsorbents for heavy-metal removal

Table 8, show the qₘₐₓ values (Langmuir qₘₐₓ) for many studies using different materials, Conditions (pH, T) vary between studies—compare carefully. Replace “(this work)” cells with your measured qₘₐₓ values when available. Engineered materials (functionalized ACFs, GO, MOFs, some modified NaAZ) can show very high qₘₐₓ values for individual ions. However, many such high values are measured under optimized lab conditions (single-ion, ideal pH, no competing cations). Reported ranges for NaAZ are broad—some studies report very high qₘₐₓ for Cu or Pb depending on synthesis and form—so directly comparing numbers requires the same test conditions (pH, ionic strength, solid/liquid ratio). aims to close the gap between high-capacity but slow microporous ion-exchangers and fast, high-surface-area materials (ACF/GO). By adding meso-porosity and small crystal size you should expect faster kinetics and better column performance vs conventional NaAZ and some bio-chars this is a key practical advantage for continuous systems. (Use your t₉₀ and MTZ results to make this point.). Zeolites (NaAZ) and many chitosan-zeolite composites have excellent cyclic stability and straightforward regeneration with NaCl brine. MOFs and some carbon materials may show decreased stability or require harsh regenerates—an important operational consideration. Bio-char and natural zeolites win on cost and scalability. Advanced materials (MOFs, GO, tuned ACFs) can be costly but useful for high-value or small-volume treatments (industrial effluents). Your method’s use of recycled alumino-silicate feed stocks and DES-assisted rapid synthesis strengthens the sustainability and scalability argument.

**Table 8:** The Comparison of NaAZ with other adsorbents for heavy-metal removal.

Adsorbent (typical form)	Representative qₘₐₓ (mg·g ⁻ ¹) — Pb² ⁺ / Cu² ⁺ / Cd² ⁺ / Ni² ⁺ / Zn²⁺	Typical conditions reported	Key advantages	Limitations/ remarks	Ref.
NaAZ zeolite — hierarchical (this work)	(qₘₐₓ) Pb: Cu: Cd: Ni: Z/n results in Table 4	batch, pH ~ 5–6, 25 °C	High cation exchange capacity, good regeneration, robust framework, tuned meso-porosity (fast kinetics)	Ion-exchange selectivity vs competing cations; micro-pore diffusion unless meso-porosity introduced	------
NaAZ/ Na-A (literature)	Pb: variable — reports from tens to several hundreds mg·g ⁻ ¹ (depends on synthesis/ form); Cu: up to hundreds mg·g ⁻ ¹ reported (some extreme values ~700–800 mg·g ⁻ ¹ in specific studies).	Many studies pH 4–6, 20–25 °C; synthesis-dependent.	Excellent ion-exchange; widely used; can be synthesized from waste streams.	Wide spread in reported qₘₐₓ — depends strongly on preparation, particle size, pretreatment. See examples.	[59,60].
Activated carbon/ activated carbon fibers (AC/ACF)	Pb: commonly 50–300 mg·g ⁻ ¹ (higher for functionalized/ACFs; reports up to ~700 mg·g ⁻ ¹ for specialized fibers). Cu: often 20–200 mg·g ⁻ ¹.	pH 4–6, 20–25 °C;	Very high surface area, tunable surface chemistry, commercial availability; high q when functionalized.	Cost (high-grade ACF), regeneration can be energy-intensive; nonselective; performance varies greatly by activation/functional groups.	[61]
Graphene oxide/ reduced GO (GO/rGO, composites)	Pb: commonly 100–300 mg·g ⁻ ¹ (examples: ~ 90–250 mg·g ⁻ ¹), some functionalized GO reports >200 mg·g ⁻ ¹. Cu/Cd similar ranges.	pH 4–6, 20–25 °C;	Very high capacities, fast kinetics, rich surface chemistry for modification.	Cost, aggregation, recovery from water (magnetic composites help), possible toxicity concerns.	[62]
Chitosan/ chitosan-based composites	Pb: wide range tens to several hundreds mg·g ⁻ ¹ (some tailored chitosan composites report >200–700 mg·g ⁻ ¹ under specific conditions). Cu/Cd also high in modified chitosan.	pH (pH 4–6).	Biopolymer (renewable), good affinity for metal cations, easy to functionalize/ crosslink.	Mechanical strength, swelling, solubility in acid, and regeneration limitations unless crosslinked/composited. .	[63]
Metal–organic frameworks (UiO-66, modified MOFs)	Pb/Cu/Cd: typically tens to few hundreds mg·g ⁻ ¹; functionalized MOFs report >200 mg·g ⁻ ¹ for Pb in some studies.	pH 3–7, 20–30 °C; MOF	Very high tunability, tailor-made binding sites, high capacity after functionalization.	Hydrolytic stability in water, cost of synthesis, scalability concerns.	[64]
Biochar//magnetic biochar	Pb: often 10–100 mg·g ⁻ ¹	pH 4–7, 20–30 °C.	Low-cost, waste-derived, facile production, good for large-scale low-cost remediation.	Lower capacity/selectivity vs advanced adsorbents; variability by feedstock/pyrolysis conditions.	[65]
Natural zeolite (clinoptilolite)	Pb: ~ 10–60 mg·g ⁻ ¹	pH 4–7, 20–25 °C.	Cheap, abundant, decent ion-exchange; robust in columns.	Lower capacity than engineered materials; selectivity influenced by competing cations.	[66]
iron oxide-commercial activated carbon nanocomposite (MV40) dye	removal of hexavalent chromium (Cr^6+^) ions and Mordant Violet 40	pH = 1.6, the contact time was 3 h and the temperature was room temperature.	Low-cost, waste-derived	---	[67]

## Conclusion

A novel template-free hydrothermal method was successfully developed for the synthesis of Na-A zeolite (NaAZ), yielding a material with excellent crystallinity, purity, and a characteristic cubic morphology. XRD analysis confirmed the formation of the LTA-type Na-A zeolite structure with sharp diffraction peaks at 2θ = 10.22°, 21.74°, 27.08°, 30.16°, and 40.88°, corresponding to the (100), (200), (321), (400), and (420) planes. XRF results showed that the material primarily consisted of Si (65 wt%), Al (17.5 wt%), and Na (6.5 wt%), consistent with the expected aluminosilicate composition. SEM micrographs revealed well-developed cubic crystals ranging from 1–3 µm, while EDX analysis confirmed the homogeneous distribution of Na, Al, Si, and O within the framework. BET analysis indicated a high specific surface area with a combination of micro- and mesopores, confirming the hierarchical porosity of the synthesized NaAZ. FTIR spectra displayed characteristic Si–O–Al stretching vibrations at 1166 and 1080 cm ⁻ ¹, as well as the D6R breathing mode near 530 cm ⁻ ¹, verifying the LTA zeolite topology. The adsorption experiments demonstrated that NaAZ exhibited outstanding affinity toward heavy metals such as Pb^2^ ⁺ , Cu^2^ ⁺ , Cd^2^ ⁺ , Ni^2^ ⁺ , and Zn^2^ ⁺ , achieving rapid equilibrium and high adsorption capacities. The enhanced performance can be attributed to its large surface area, high cation-exchange capacity, and accessible pore network. In summary, the synthesized Na-A zeolite possesses the desired structural, morphological, and chemical features that make it an efficient, low-cost, and environmentally friendly adsorbent for heavy metal removal and other wastewater treatment applications. Future work will focus on regeneration studies and performance evaluation under real wastewater conditions.

## Supporting information

S1 File (XLSX)
